# Exercise as a Useful Intervention to Reduce Alcohol Consumption and Improve Physical Fitness in Individuals With Alcohol Use Disorder: A Systematic Review and Meta-Analysis

**DOI:** 10.3389/fpsyg.2021.675285

**Published:** 2021-07-07

**Authors:** David T. Lardier, Kathryn E. Coakley, Kelley R. Holladay, Fabiano T. Amorim, Micah N. Zuhl

**Affiliations:** ^1^Department of Individual, Family and Community Education, College of Education and Human Sciences, University of New Mexico, Albuquerque, NM, United States; ^2^Department of Psychiatry and Behavioral Sciences, University of New Mexico School of Medicine, University of New Mexico, Albuquerque, NM, United States; ^3^Department of Individual, Family and Community Education, University of New Mexico, Albuquerque, NM, United States; ^4^College of Health Sciences, Jacksonville University, Jacksonville, FL, United States; ^5^Department of Health Education and Sports Sciences College of Education and Human Sciences, University of New Mexico, Albuquerque, NM, United States; ^6^School of Health Sciences, Central Michigan University, Mount Pleasant, MI, United States

**Keywords:** alcohol use disorder, alcohol dependence, binge drinking, exercise, physical activity, randomized controlled trials

## Abstract

**Objective:** This meta-analysis and systematic review examined the effects of exercise interventions on alcohol consumption and binge drinking in individuals with alcohol use disorder (AUD).

**Data sources:** PubMed, Web of Science, Google Scholar, SPORTDiscus, and ERIC databases.

**Study Inclusion and Exclusion Criteria:** Peer-reviewed randomized controlled trials published in English between 1970 and 2021. All studies compared exercise (Ex) and treatment as usual (TAU) to TAU in adults with an alcohol-related diagnosis. All forms of exercise interventions were included (e.g., aerobic exercise, yoga, resistance exercise, etc.).

**Data Extraction:** Preferred Reporting Items for Systematic Reviews and Meta-Analyses Protocols standard and the Meta-Analyses and Systematic Reviews of Observational Studies guidelines were followed. Risk of bias was assessed using the Cochrane risk-of-bias tool as described by the Cochrane Handbook for Systematic Reviews and Interventions.

**Results:** The literature searches retrieved a combined 2527 studies, with 1,034 studies screened after removal of duplicates and 973 (94%) rejected after reviewing titles and abstracts. Full-text review was performed on 61 studies, with seven studies meeting inclusion criteria for qualitative and meta-analysis. Across seven studies (*n* = 492 participants), a significant effect (Z-value = −3.37; g = −0.30; 95% CI [−0.50—−0.09]; *p* = 0.001) was found for Ex+TAU on drinking volume. There was no effect of Ex+TAU on binge drinking. The effect of Ex+TAU on physical fitness (VO_2_max, ml•kg^−1^•min^−1^) was significant (Z-score = 3.70; g = 0.64; 95% CI [0.19–1.08]; *p* < 0.001).

**Conclusions:** Exercise interventions may decrease alcohol consumption and improve fitness and can be an effective adjunctive treatment for individuals with alcohol-related diagnoses including AUD.

## Introduction

Alcohol use disorder (AUD) is a chronic condition affecting nearly 283 million people aged 15 years and older worldwide and 14.4 million adults ages 18 years and older in the United States (World Health Organization, 2010; National Institute on Alcohol Abuse Alcoholism, 2017). The Diagnostic and Statistical Manual—Version 5 (DSM-5) defines AUD as a problematic pattern of alcohol use leading to clinically significant impairment or distress (American Psychiatric Association, 2013). The DSM-5 further describes AUD as a cluster of behavioral and physical symptoms that can include withdrawal, tolerance, and craving, which can lead to both alcohol abuse and dependence (American Psychiatric Association, 2013). Individuals with AUD may also binge drink, defined as consuming five or more standard drinks over 2 hours for men, or four or more over 2 hours for women (Centers of Disease Control and Prevention, 2020). Binge drinking is the most common, costly, and deadly pattern of alcohol use in the United States (Centers of Disease Control and Prevention, 2020).

Individuals with AUD suffer adverse social, occupational, and health consequences, including high mortality (Carvalho et al., 2019; Witkiewitz et al., 2019). In 2016, the estimated number of deaths associated with the harmful use of alcohol was ~3 million worldwide (or 5.3% of all deaths) (World Health Organization, 2010; Carvalho et al., 2019). Health consequences of heavy chronic alcohol use includes direct toxic effect on the liver and gastrointestinal tract and its association with heart disease and cancer. AUD often co-occurs with other mental health disorders including depression, anxiety, or psychoses (Castillo-Carniglia et al., 2019). Even in the absence of other mental health comorbidities, chronic alcohol intoxication may impair cognitive function leading to a progressive decline of behavioral control. Alcohol consumption is also frequently associated with the use of tobacco smoke and other psychoactive substances (World Health Organization, 2010; National Institute on Alcohol Abuse Alcoholism, 2017).

AUD is complex, with a host of underlying mechanisms and associated symptoms. The root cause of AUD likely results from a combination of genetic, environmental, and lifestyle factors (Carvalho et al., 2019; Cabe et al., 2020). Comorbid conditions associated with AUD may include depression, anxiety, and low self-efficacy (American Psychiatric Association, 2013), which all appear to be predictors of alcohol consumption and relapse (Carvalho et al., 2019; Castillo-Carniglia et al., 2019). The complexities of AUD pose a challenge to maintain long-term sobriety in 50–80% of individuals (Jin et al., 1998; Moos and Moos, 2006). First year relapse rates range upwards to 90% among those who seek treatment (Moos and Moos, 2006).

Treatment approaches have been explored in attempts to deconstruct the complexness of AUD. These have included behavioral therapies, such as cognitive behavioral therapy, contingency management, motivational interviewing, and behavioral therapies combined with pharmacotherapy (Jhanjee, 2014). Even more recently, tailored treatment approaches using genetic testing have been studied and developed to treat underlying mechanisms (Seneviratne and Johnson, 2015). Despite advancements, combined and tailored treatments have provided little effectiveness beyond behavioral treatment alone, with relapse remaining high and pharmacological therapeutic interventions having unwanted side effects (Maisto et al., 2000; Miller et al., 2001; Berglund et al., 2003; Hallgren et al., 2017a). Moreover, many of the treatment options available require extensive time and money. A recent meta-analysis showed that 50% of people with AUD achieved remission 16 years after the onset of the disorder, which highlights the challenges in meeting the therapeutic needs of this population (Fleury et al., 2016). Novel adjunctive interventions are needed to address AUD, alcohol abstinence, complex psychopathological vulnerabilities related to AUD such as alcohol cravings (Frascella et al., 2010; Di Nicola et al., 2015), comorbid health problems commonly associated with AUD including depression and anxiety (Hallgren et al., 2014a; Åhlin et al., 2015), and cardiometabolic risks (Vancampfort et al., 2016).

Exercise training is well-known to promote a host of positive physical adaptations including the prevention and treatment of cardiovascular and metabolic diseases (Ruegsegger and Booth, 2018). More recently, studies have identified exercise training as a non-invasive and non-pharmacological therapy for neurological diseases including neurodegenerative diseases (e.g., Parkinson's and Alzheimer's disease) (Ahlskog et al., 2011), depression (McKercher et al., 2014), and anxiety (Greenwood et al., 2012; Furzer et al., 2021). Exercise has also been suggested as a potential treatment for substance use disorders (SUD) (Lynch et al., 2013; Cabe et al., 2020; Furzer et al., 2021) and has been incorporated into alcohol treatment programs for over 40 years (Sinyor et al., 1982). Activities have included sports (e.g., basketball), jogging and calisthenics, yoga, and targeted exercise with intensity and duration benchmarks (Gür and Gür, 2020). Reported outcomes have been improvements in cardiovascular fitness and mood-related changes with only a small number of studies reporting alcohol consumption measures (Cabe et al., 2020).

Recently published systematic reviews have examined the impacts of exercise training on treatment for AUD (Hallgren et al., 2017a; Cabe et al., 2020; Gür and Gür, 2020); exercise may serve many of the underlying symptoms associated with AUD including mental health. However, the impact of exercise on alcohol use, including binge drinking, is limited. Therefore, the aim of this meta-analysis is to examine the treatment effects of exercise training on measures of alcohol consumption and binge drinking in individuals with diagnosed AUD, or in the extant literature described as experiencing alcohol dependence, alcohol abuse, and harmful drinking behavior. A secondary aim was to evaluate changes in cardiovascular fitness among this population.

## Methods

### Inclusion and Exclusion Criteria

Article searches were conducted in March 2021. Studies eligible for inclusion in this meta-analysis met the following criteria: (1). randomized controlled trial (RCT) that compared exercise and treatment as usual (Ex+TAU) to treatment as usual (TAU); (2). exercise intervention was described and included more than one session; (3). drinking outcomes were reported and included alcohol consumption, binge drinking, and/or cravings; (4). study population included adults with an alcohol-related diagnosis (defined in Section Study Participants); (5). published in English in a peer-reviewed journal between 1970-2020. The following studies were excluded: non-RCT studies (e.g., cross-sectional, cohort studies), studies including a single session of exercise, and/or studies that did not report alcohol consumption. All forms of exercise were eligible for inclusion (e.g., aerobic exercise, yoga, resistance exercise, etc.).

### Study Participants

Adult participants, at least 18 years of age, in inpatient and outpatient treatment programs, community programs, or other public health interventions were included in this systematic review. College students were also included. All participants met the criteria for AUD, alcohol abuse or alcohol dependence defined by the DSM, harmful use of, or dependence on alcohol per ICD-9 or ICD-10 criteria, or hazardous drinking per Alcohol Use Disorders Identification Test (AUDIT) score.

### Outcome Measures

All studies included at least one primary outcome included in the meta-analysis: number of standard drinks per week and/or binge drinking episodes. An attempt was made to conduct a meta-analysis for alcohol cravings, but only two RCTs were located for analysis. Physical fitness, recorded as maximal oxygen consumption (VO_2_max in ml•kg-1•min-1), was also assessed as a secondary outcome.

### Data Sources

All study authors searched PubMed, Web of Science, Google Scholar, SPORTDiscus, and ERIC databases in March 2021 using key terms: [(exercise OR “aerobic exercise” OR “physical activity” OR yoga OR “resistance exercise”) AND (“alcohol abuse” OR “alcoholism” OR “alcohol addiction” OR “binge drinking” OR “heavy drinking” OR “alcohol dependence” OR “alcohol use disorder”)].

### Study Selection

Once all articles were retrieved from individual databases, results were compiled, and duplicates were removed. Following the removal of duplicate studies, authors screened titles and abstracts to determine eligible articles. Full text articles were included, and a final selection of articles was established based on the consensus of all author reviewers. An audit trail was maintained throughout the entire process which allowed the research team to refer to and examine discrepancies. Discrepancies were discussed until agreement was reached.

### Data Extraction and Management

Two authors extracted data from each included study using a data collection form. Table 1 displays data extraction information including study title, population studied, number of participants in the study and percent male, age of study participants, the exercise intervention, outcomes assessed, results, and conclusions.

**Table 1:** Summary of included studies and results.

**References**	**Title**	**Population**	***n***	**%Male**	**Age [mean (SD)]**	**Exercise intervention**	**Outcome(s)**	**Results**	**Conclusions**
Jensen et al. (2019)	Physical exercise in the treatment of alcohol use disorder (AUD) patients affects their drinking habits: A randomized controlled trial	AUD or alcohol abuse or dependence (DSM criteria)	105	71.4	Women (*n =* 30): 51.1 ± 11 Men (*n =* 75): 43 ± 12	Group or individual walking/running for two 60-min sessions per week over 24 weeks.	Number of standard drinks (30 days)	Significant reduction in standard drinks (30 days) in all groups: 221 ± 219 at baseline to 43 ± 87 6-months post-intervention (*p <* 0.0001). No difference between groups.	Including physical exercise in the treatment of alcohol disorder patients will affect drinking habits. Form of exercise (individual vs. group) does not make a difference.
Weinstock et al. (2020)	Randomized Clinical Trial of Exercise for Non-treatment seeking adults with alcohol use disorder	AUD	66	39.4	MO (*n =* 33): 35.9 ± 12.0 MI+CM (*n =* 33): 33.8 ± 8.0	Weekly contingency management (CM) exercise contracting sessions and motivational interviewing for 16 weeks; 4-month YMCA membership.	Number of standard drinks (7 days) Number of binge drinking episodes (7 days)	Significant reduction in weekly total standard drinks and binge episodes from baseline to mid-treatment and post-treatment in both groups. No difference between groups.	All participants, regardless of group assignment, reduced their drinking by about 50% at mid-treatment and maintained reductions at post-treatment including reduction of binge drinking episodes from an average of 2–3 to 1 per week. Increases in exercise were not associated with reductions in drinking.
Roessler et al. (2017)	Exercise as adjunctive treatment for alcohol use disorder: A randomized controlled trial	Harmful use of or dependence on alcohol (ICD-10)	175	68.6	45 ± 11.3; range 21-70	Group or individual walking/running for two 60-min sessions per week over 24 weeks.	Number of standard drinks (30 days)	Moderate physical activity level had a protective effect on drinking behavior compared to low PA levels. No significant differences between treatment groups. Amount of alcohol consumed in intervention groups decreased by 4% for each increased exercising day.	This study supports existing evidence that physical activity may be effective as adjunctive treatment for AUD. Moderate level PA was protective against excessive drinking at follow-up.
Brown et al. (2014)	A preliminary, randomized trial of aerobic exercise for alcohol dependence	Alcohol dependence (DSM-IV)	49	55	44.37 ± 10.75	12 weekly supervised moderate intensity group aerobic exercise sessions.	Number of standard drinks/day (90 days) Percent heavy drinking days (90 days)	Significant decrease in drinking days and heavy drinking days in exercise group vs control group during treatment; not maintained at 12-week follow-up.	Group aerobic exercise intervention reduced alcohol use compared to brief advice to exercise. Amount of moderate-intensity exercise did not fully account for decreases.
Hallgren et al. (2014b)	Yoga as an adjunct treatment for alcohol dependence: a pilot study	Alcohol dependence (DSM-IV)	18	Not reported	Not reported	Weekly yoga session (1.5 h) for 10 weeks.	Number of standard drinks (7 days)	Significant decrease in weekly alcohol consumption in both groups but not statistically different between groups.	Yoga intervention resulted in a larger (but not significantly so) reduction in weekly drinks compared to treatment as usual. Improvements were demonstrated in both groups.
Weinstock et al. (2014)	Exercise as an intervention for sedentary hazardous drinking college students: A pilot study	Hazardous drinking (AUDIT >8); college students	31	35.5	MET (*n =* 15): 20.1 ± 1.2 MET+CM (*n =* 16): 21.0 ± 2.3	One 50-min Motivational Enhancement Therapy (MET) session focused on exercise + 8 weeks of contingency management (CM) for adhering to specific exercise activities.	Number of standard drinks (7 days) Number of heavy drinking days (60 days)	No changes in total drinks per week or heavy drinking days in either group. No significant difference between groups. MET alone resulted in greater reduction in heavy drinking episodes.	No significant changes or differences in drinking behavior over time or by treatment condition over time.
Murphy et al. (1986)	Lifestyle modification with heavy alcohol drinkers: effects of aerobic exercise and meditation	Male college students identified as high volume drinkers (>1.5 drinks/days or 45 drinks/month)	48	100	Runners (*n =* 13): 24.9 Meditators (*n =* 14): 25.0 Controls (*n =* 16): 24.5	Running group - 70 min supervised group running sessions 3 times/week for 8 weeks Meditation group—supervised group meditation session 3 times/week + 20 min of meditation twice a day, every day on own for 8 weeks	Mean weekly ethanol consumption	At weeks 3–10, mean ethanol consumption was lower in the running group vs. control group. Subjects in running group reduced consumption by 60% from baseline. No significant differences between meditation group and control group.	A regular program of aerobic running leads to a significant reduction in alcohol consumption for subjects who are heavy social drinkers (~14 fewer drinks per week). A group-based program seems to facilitate compliance.

### Risk of Bias/Quality Assessment

Publication bias was assessed using the Cochrane risk-of-bias tool as described by the Cochrane Handbook for Systematic Reviews and Interventions (Higgins et al., 2019). Each study was evaluated on five domains: (1). The randomization process; (2). The study deviation from the intervention; (3). Missing outcome data; (4). Adequate measurement of outcome data; and (5). Bias in the selection of the reported results. An overall bias assessment was made regarding each study.

### Statistical Analyses

Using random–effects models, meta–analyses were conducted using Meta Essentials for Microsoft Excel *(*version 1.5) (van Rhee et al., 2015). Weighted mean differences with 95% confidence intervals (95% CIs) between pre- and post-exercise intervention on primary outcomes (weekly alcohol use and binge drinking episodes) and secondary outcome (VO_2_max) measures for both intervention and control groups were divided by pooled standard deviation. Monthly alcohol use measures were converted to weekly alcohol use. Binge drinking was reported as drinking episodes or heavy drinking days. Hedges' *g* was used to evaluate the magnitude of the overall effect size with weighted mean differences of small (0.2–0.5), moderate (0.5–0.8), and large (>0.8) (Hedges and Olkin, 2014). The small number of included studies (<10) limited the ability to conduct subgroup analyses (Thompson and Higgins, 2002). Covariates included age, baseline drinking measures, gender, and duration of the exercise program (in weeks). Other exercise covariates such as exercise intensity, type, and adherence were considered but lack of intervention descriptions, and thus sample size, excluded these analyses. Heterogeneity was assessed using the I^2^ index with low heterogeneity (*I*^2^ = 0–25%), moderate heterogeneity (*I*^2^ = 26–50%), substantial heterogeneity (*I*^2^ = 51–75%), and considerable heterogeneity (*I*^2^ = 76–100%) (World Health Organization, 2010). The Egger Regression was used to adjust for publication bias (American Psychiatric Association, 2013). Statistical significance was set to *p* < 0.05.

## Results

### Search Results

**Figure 1:**
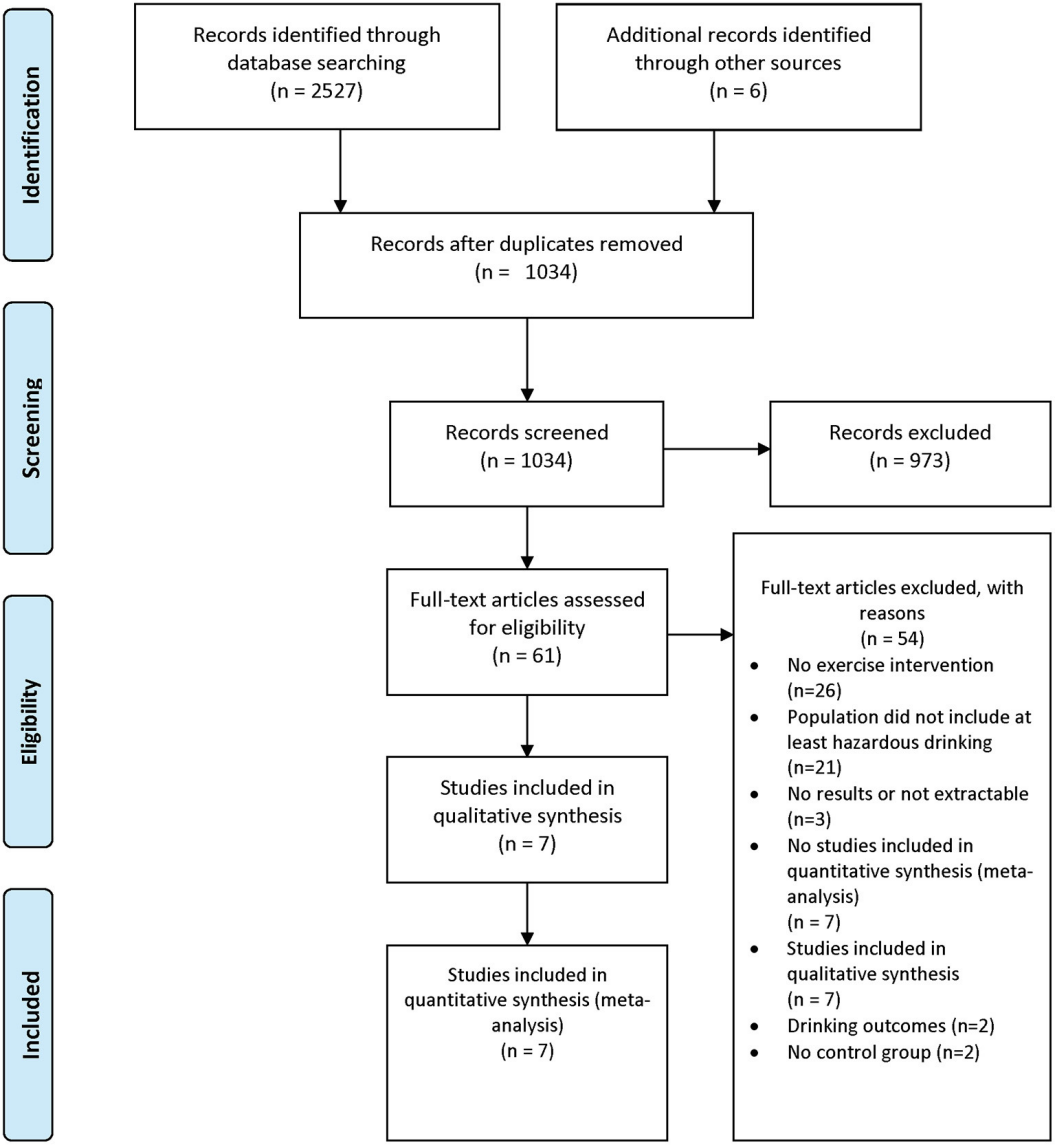
PRISMA flow diagram. PRISMA, preferred reporting items for systematic reviews and meta-analyses. This PRISMA diagram contains public sector information licensed under the Open Government Licence v3.0. (Adapted From: Moher et al., 2009).

The literature searches retrieved a combined 2,527 studies. The flow diagram of identification, screening, eligibility, and inclusion of studies is shown in Figure 1. After the deletion of duplicates, 1,034 studies were screened, and 973 (94%) were rejected by reviewing titles and abstracts. Full-text review was performed on the remaining 61 studies. Of these studies, 54 (88.5%) were excluded for the following reasons: 26 did not include an exercise intervention, 21 did not include a population with at least hazardous drinking, three did not include results or had results that were not extractable, two did not include drinking-related outcomes, and two did not have a control group. Seven studies were included in the final qualitative and meta-analysis.

Across the seven studies, a total of 492 participants were included (mean sample size = 70.3 ± 49.9). On average, 61.7% of participants were male. The average age of participants ranged from 20 to 51 years of age. Three studies included participants with alcohol dependence (Brown et al., 2014; Hallgren et al., 2014b; Roessler et al., 2017), two included participants with hazardous/high-volume drinking (Murphy et al., 1986; Weinstock et al., 2014), one included participants with AUD or alcohol dependence (Jensen et al., 2019), and one included participants with AUD only (Weinstock et al., 2020). All included studies examined number of standard drinks over a 1-week or 1-month period (*n* = 7) and four of the seven studies (57%) examined binge drinking episodes. Four studies were conducted among treatment seeking adults (Brown et al., 2014; Hallgren et al., 2017b; Roessler et al., 2017; Jensen et al., 2019), and three studies recruited non-treatment seeking adults (Murphy et al., 1986; Weinstock et al., 2014, 2020).

### Exercise Interventions

The exercise interventions ranged from 8 to 24 weeks (mean = 14.6 ± 6.5 weeks). Duration of exercise ranged from 90 min (Hallgren et al., 2014b) to 150 min per week (Weinstock et al., 2014, 2020) with an average of 127 ± 20 min across the seven studies. Four of the seven studies included moderate-intensity aerobic training (Brown et al., 2014; Roessler et al., 2017; Jensen et al., 2019; Weinstock et al., 2020), one included low to moderate aerobic training (Weinstock et al., 2014), one included yoga (low intensity) (Hallgren et al., 2014b), and one included running or walking without a defined intensity (Murphy et al., 1986).

Of the six aerobic exercise studies, two studies used both group and individual exercise study arms (Roessler et al., 2017; Jensen et al., 2019). A running program was tested in three studies, which consisted of supervised running two (Roessler et al., 2017; Jensen et al., 2019) or three times per week (Murphy et al., 1986). One exercise intervention was designed to meet aerobic exercise recommendations set by the American College of Sports Medicine (ACSM) and included 20–40 min at 50–69% intensity (based on maximum heart rate) up to three times per week (Brown et al., 2014). Another study did not directly implement the exercise intervention but combined an exercise program with contingency management (CM). The weekly CM sessions encouraged the participants to complete various types of exercise (treadmill walking or jogging) or an instructor-led exercise class (Weinstock et al., 2014). Three studies used incentives (vouchers for prizes) to motivate participants to complete exercise (Brown et al., 2014; Weinstock et al., 2014, 2020). Alcohol measures were assessed prior to beginning treatment and were re-examined at the end of the exercise intervention (post testing timeframes were not discussed in the included studies). Follow-up assessments conducted after end-of-treatment measurements were not included in the analysis.

### Treatment as Usual Interventions

Four of the studies included participants enrolled in an outpatient clinic or day treatment program for alcohol use (Brown et al., 2014; Hallgren et al., 2014b; Roessler et al., 2017; Jensen et al., 2019). In these studies, treatment as usual included cognitive behavioral therapy (Brown et al., 2014; Hallgren et al., 2014b; Roessler et al., 2017; Jensen et al., 2019), motivational interviewing (Hallgren et al., 2014b; Roessler et al., 2017; Jensen et al., 2019), family therapy (Roessler et al., 2017; Jensen et al., 2019), and/or pharmacologic management (Hallgren et al., 2014b; Roessler et al., 2017; Jensen et al., 2019). Three studies included community-dwelling participants who were not receiving treatment for alcohol use (Murphy et al., 1986; Weinstock et al., 2014, 2020). In these studies, the control group received a YMCA membership only (Weinstock et al., 2020), a brief one-time wellness intervention for increasing exercise adherence that did not address drinking (Weinstock et al., 2014), and instruction to keep a daily journal of behavior (Murphy et al., 1986).

### Publication Bias

The Cochrane risk-of-bias tool was used to assess bias among included studies. Randomization was reported in all studies, and the process of randomization was low risk in five studies (Hallgren et al., 2014b; Weinstock et al., 2014, 2020; Roessler et al., 2017; Jensen et al., 2019), and presented some concern in two studies due to large baseline differences among groups, which suggested a problem with the randomization process (Murphy et al., 1986; Brown et al., 2014).

There was also a lack of blinding in all seven studies. Participants selected for the exercise arms in each study were likely aware of their assigned intervention. In addition, the individuals who delivered or supervised the exercise intervention were aware of group assignment. However, it is unlikely that this presented actual risk of bias. The risk of bias due to missing outcome data was assessed as low in four studies (Murphy et al., 1986; Brown et al., 2014; Weinstock et al., 2014, 2020), and was somewhat concerning in three studies (Hallgren et al., 2014b; Roessler et al., 2017; Jensen et al., 2019). Across studies, there was some degree of missing data from participant dropout, which may have introduced bias in the outcome. Risk of bias due to outcome measures was high in one study (Murphy et al., 1986) because of the possible inaccuracy in the method for collecting drinking behavior. All other studies were low risk. The risk of bias in selection of reported results was uncertain due to lack of pre-specified analysis plans. Of the included studies, two had previously published methodology papers that included protocols for statistical analyses (Brown et al., 2014; Roessler et al., 2017). In summary, there was some concern for risk of bias in selected studies.

### Effect of Exercise + TAU on Drinks per Week

**Figure 2:**
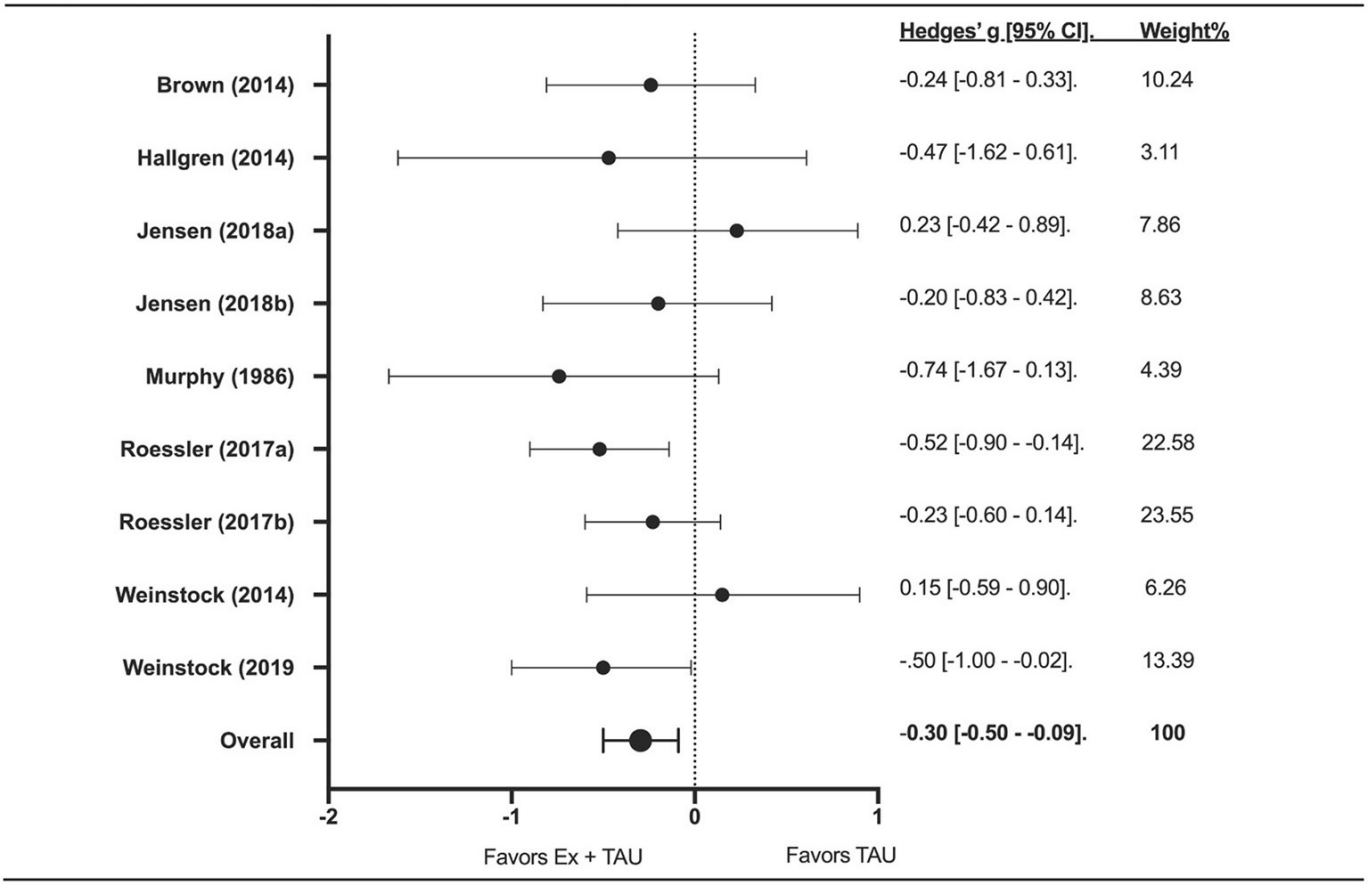
The forest plot about the effect of exercise on drinking volume.

All studies compared Ex+TAU vs. TAU on weekly drinking volume and/or binge drinking. Two studies included three groups in their intervention (e.g., individual exercise, group exercise, and control). Therefore, nine effect sizes were calculated (Figure 2). The effect of Ex+TAU on weekly drinking volume was significant (Z-value = −3.37; g = −0.30; 95% CI [−0.50–−0.09]; *p* = 0.001) indicating a small effect in favor of Ex+TAU compared to TAU alone. The *I*^2^ index showed 0.00% heterogeneity.

### Effect of Exercise + TAU on Binge Drinking Episodes

**Figure 3:**
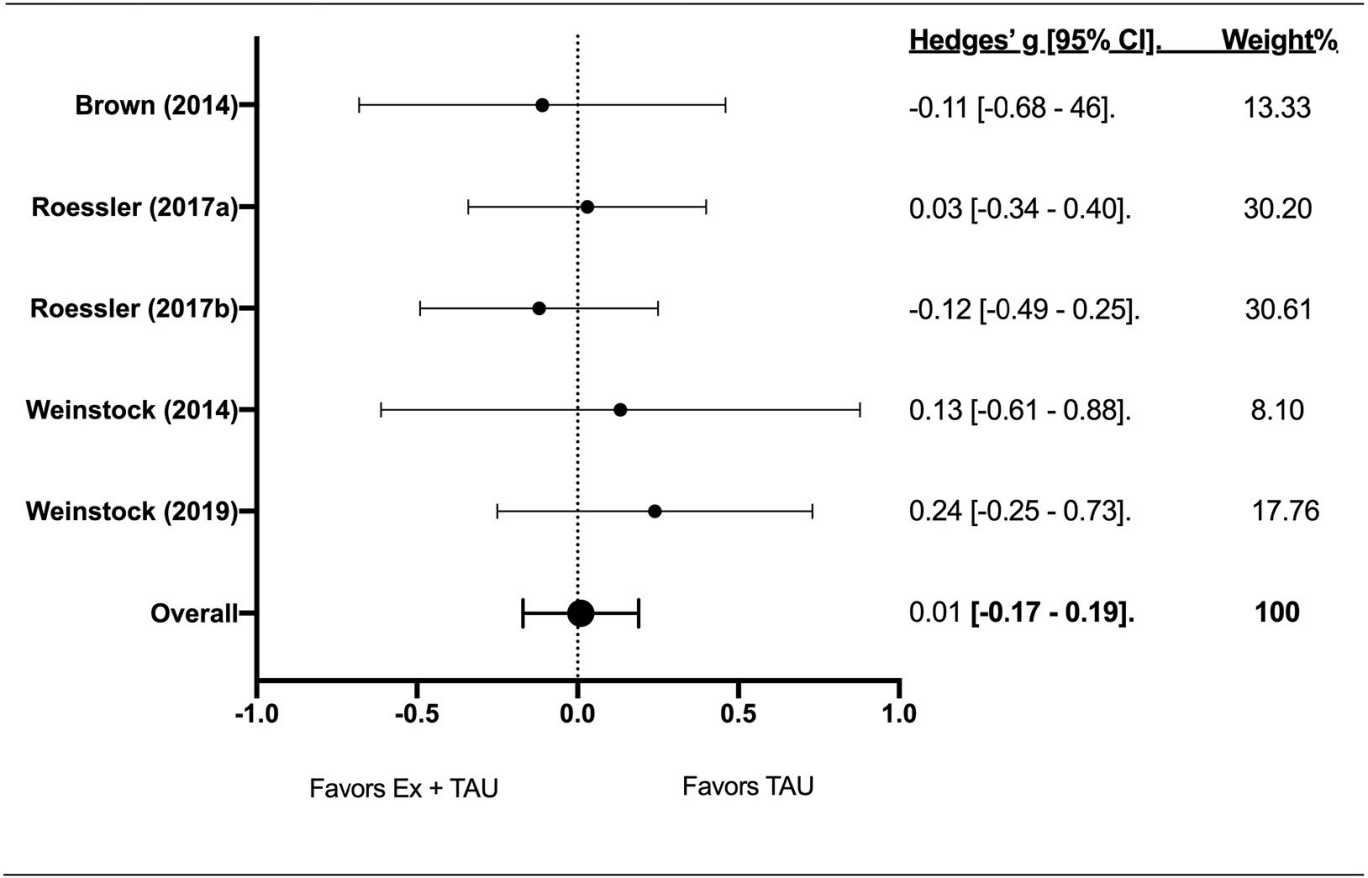
The forest plot about the effect of exercise on binge drinking.

Five effect sizes were calculated for binge drinking episodes among four studies (Figure 3). The total sample size among studies was 372. The effect of Ex+TAU on binge drinking episodes was not significant (Z-score = 0.14; g = 0.01; 95% CI [−0.17–0.19]; *p* = 0.89) indicating no effect in favor of TAU. The *I*^2^ index showed 0.00% heterogeneity.

### Effect of Exercise + TAU on Physical Fitness

**Figure 4:**
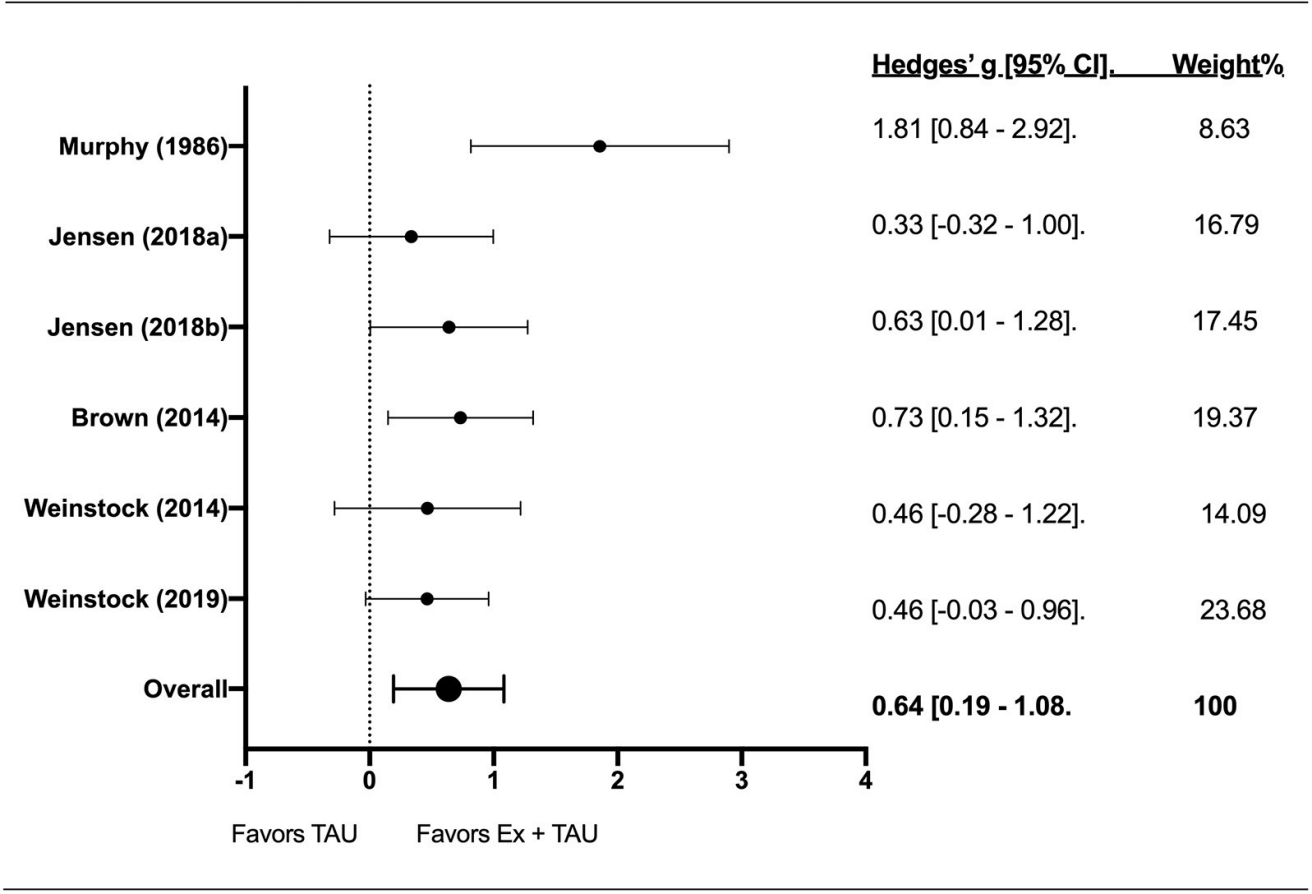
The forest plot about the effect of exercise on physical fitness.

Six effect sizes were calculated for physical fitness (VO_2_max, ml•kg^−1^•min^−1^) among five studies (Figure 4). The mean baseline VO_2_max was 30.60 ± 5.03 and 33.17 ± 3.49 ml•kg^−1^•min^−1^ for TAU and Ex+TAU, respectively. Mean end of treatment VO_2_max was 32.12 ±3.65 ml•kg^−1^•min^−1^ for TAU and 36.27 ± 2.40 ml•kg^−1^•min^−1^ for Ex+TAU. The effect of Ex+TAU on physical fitness was significant [Z-score = 3.70; g = 0.64; 95% CI [0.19–1.08]; *p* < 0.001]. The *I*^2^ index revealed 31% heterogeneity.

### Covariate Analyses

Study participant age, gender, or baseline alcohol consumption did not influence outcomes. Similarly, there was no moderation effect of exercise duration (in weeks) on drinking outcomes.

## Discussion

This systematic review and meta-analysis are among the first to examine the pooled effects of exercise interventions on alcohol consumption and binge drinking episodes in those with AUD, or as prior literature described as alcohol abuse, alcohol dependence, or harmful drinking. Seven randomized controlled trials met the inclusion criteria including a combined 492 adults. Pooled data indicated exercise interventions significantly reduced weekly drinking volume but had no impact on binge drinking episodes. Exercise interventions also significantly improved cardiovascular fitness.

Alcohol-related disorders are significant international public health concerns (Carvalho et al., 2019). Recent investigations suggest at least 10% of adults who receive substance use treatment experience deterioration or symptom exacerbation during treatment or soon thereafter (Shaw et al., 1997; Moos et al., 2001, 2002). There are several psychosocial interventions for the treatment of AUD in adults. These psychosocial interventions include coping skills training, relapse prevention, motivation intervention, motivational enhancement therapy (Carroll and Onken, 2005; Jensen et al., 2011; Gates and Albertella, 2016; Knapp et al., 2016), recovery support interventions (Powell et al., 2019), and group-based formats (Wendt and Gone, 2017). And while innovations in AUD treatment have occurred over the past 30 years, adjunctive treatments such as mindfulness, wellness, and exercise may now be included in addition to traditional psychotherapies (Simpson and Sells, 1982; Mattson et al., 1993; Cabe et al., 2020).

Despite these innovations, barriers to treatment for adults in this vulnerable group remain. Mental health and physical co-morbidities create complexities to the effective treatment of AUD (Mojtabai et al., 2014; Lipari et al., 2016; Cabe et al., 2020; Furzer et al., 2021). The extant literature agrees that there is a gene-environment interaction, influencing alcohol cravings and maintenance of long-term sobriety (Kendler et al., 2012; Mitchell et al., 2013; Carvalho et al., 2019). Researchers concur that treatments that combine multiple approaches are most effective for treating AUDs and helping individuals maintain sobriety (Jhanjee, 2014; Witkiewitz et al., 2019). Exercise training has been identified as an adjunctive treatment modality for AUD (Hallgren et al., 2017a; Gür and Gür, 2020; Furzer et al., 2021), though a better understanding of how exercise improves outcomes is still needed (Cabe et al., 2020).

This meta-analysis contributes to the limited yet expanding literature, finding a significant reduction in drinking volume of approximately one-third a standard deviation for exercise compared with control conditions (g = −0.30). While a smaller effect size, overall, these findings are clinically meaningful, displaying subtle improvements in drinking volume reductions based on participation in physical exercise programs. Findings among seven studies are reliable as evident by low heterogeneity, indicating consistency for the positive effect of exercise on drinking volume. As one of few studies to specifically examine the pooled treatment effects of exercise interventions on alcohol consumption and binge drinking episodes in those with AUD, our findings contrast with recent meta analyses (Hallgren et al., 2017a; Gür and Gür, 2020). Thompson et al. reported no clear evidence for an effect of exercise on alcohol consumption based on two studies, but included non-RCTs and participants with any SUD, not just AUD (Thompson et al., 2017). The authors did, however, report a significant effect of exercise on the prevention of alcohol initiation. Gür and Gür found that exercise did not reduce alcohol consumption in adults with AUD but did improve physical fitness and mental health (Gür and Gür, 2020). The authors included non-RCTs, did not include studies published prior to 2000, and included only five studies in the meta-analysis of alcohol consumption. Hallgren, Vancampfort, Giesen, Lundin, Stubbs (Hallgren et al., 2017a) found that exercise did not reduce daily alcohol consumption or AUDIT total scores in adults with AUD. Although they reported a positive reduction on weekly alcohol consumption (*p* = 0.04), this result was not significant after adjustment for publication bias. The authors included individuals with AUD only, included studies with single bouts of exercise as the intervention, and included studies without a control group. Unlike these three meta-analyses, the current meta-analysis included RCTs that compared a control group with exercise training interventions (several sessions of exercise) among individuals with AUD, or reported as alcohol abuse, alcohol dependence, or harmful drinking behaviors.

In addition, the effect of exercise on binge drinking episodes was examined in this study with no added benefit detected among four pooled studies (generating five effect sizes). Binge drinking behavior did decrease among both TAU and Ex+TAU groups in each study, but no added effect of exercise was observed. One possible explanation is the positive association between physical exercise and binge drinking that has been previously reported in the literature (Musselman and Rutledge, 2010). Individuals may seek to reward exercise accomplishments with episodes of heavy drinking, which has been documented among university students and athletes (Nelson and Wechsler, 2001; Vickers et al., 2004). Establishing a link between exercise and binge drinking in early adulthood may extend into later life; therefore, exercise may counteract the benefits of therapeutic treatment for these individuals. Binge drinking in college and early adulthood is a risk factor for continued alcohol misuse (Jennison, 2004). This may highlight the importance of assessing the connection between physical activity and drinking behavior prior to enrollment in an adjunctive exercise program. However, exercise has been shown to reduce urges to drinking among heavy alcohol consumers (Taylor et al., 2013; Brown et al., 2016).

Cardiovascular fitness improvement measured by VO_2_max is a useful marker of exercise intervention effectiveness. Previously sedentary individuals who adhere to exercise should experience fitness gains. As such, Ex+TAU had a significant effect on VO_2_max in the current analysis, which aligns with results reported by Gür and Gür (2020). Low cardiovascular fitness has been reported among those suffering from mental health concerns, and improvements in fitness after participation in exericse has been shown to mitigate symptoms of depression and anxiety (Heggelund et al., 2020; Miller et al., 2020). AUD is highly linked to both anxiety and depression and, therefore, exercise-related changes in mood state may explain the benefits of exercise in AUD (Lai et al., 2015). This conclusion cannot, however, be evaluted in the current study and should be pursued in future research investigations.

The findings from this meta-analysis should be interpreted with caution. Our analyses were limited to seven RCT studies with two studies having both individual and group treatment interventions. Future meta-analyses may consider separating individual and group-based exercise interventions and include crossover studies. Furthermore, our results only captured the effects of exercise interventions on weekly drinking volume and binge drinking episodes. Weekly drinking volume was assessed using a variety of methods. Future studies should standardize measures of alcohol consumption and consider including alcohol abstinence, alcohol abstinence self-efficacy (DiClemente et al., 1994; Glozah et al., 2015), heavy drinking days, relapse, and alcohol cravings (Flannery et al., 1999). In addition, the effect sizes identified in this meta-analysis were small. Future research should focus on implementing rigorous RCTs to create and adapt individual and group-based exercise interventions in AUD and risky drinking populations. Moreover, due to limitations in study designs described above, the effect of exercise on AUD should be considered with caution until more substantive and rigorous studies are conducted.

## Conclusions

Our findings provide important evidence that exercise interventions can be an effective adjunctive therapy for AUD, specifically reducing weekly drinking volume and improving physical fitness. The inclusion of individuals with alcohol-related disorders in the current analysis along with examining drinking behavior adds to the still limited data regarding the benefits of exercise. Future studies are needed to clarify the mechanisms driving the reduction in drinking among individuals with AUDs. The potential benefits of exercise should also be explored among adolescents with AUD. Moreover, researchers should explore the impact of exercise combined with validated and efficacious psychotherapeutic treatments (e.g., cognitive behavioral therapy, dialectical behavioral therapy) (Cabe et al., 2020). Such research will provide crucial information on exercise as a useful and adaptive adjunctive treatment for AUD and other alcohol use issues (Cabe et al., 2020).

The utility of exercise as an adaptive add-on treatment to AUD may help provide additional treatment options for people with other complex behavioral addictions (Frascella et al., 2010; Marazziti and Baroni, 2014), which are often unmet in traditional treatment programs (Di Nicola et al., 2015). For instance, studies have outlined psychopathological vulnerabilities underlying alcohol cravings through three psychobiological pathways: *reward cravings* (i.e., the desire for rewarding, stimulating and/or enhancing effects of alcohol), *relief cravings* (i.e., desire for reduction of tension or arousal), and *obsessive cravings* (i.e., lack of control over intrusive thoughts about drinking resulting impaired functioning) (Verheul et al., 1999; Di Nicola et al., 2015). Assessing cravings typologies alongside exercise as an adjunctive treatment could provide useful differential treatment options (Hallgren et al., 2017a,b; Davis-Martin et al., 2021).

Little remains known on the optimal exercise mode, frequency, duration, and intensity needed to treat AUDs, as well as reduce the heightened cardiometabolic risks associated with AUDs, lower depression and improve mood states. Yet, results from this meta-analysis call on future research to integrate epidemiology with neuroscience and neuroimaging to better address the underlying mechanisms behind findings in this manuscript related to exercise, physical fitness improvements, better neurovascular prefrontal cortex (PFC) functioning, increased behavioral control, and reductions in overall alcohol use. In addition, results from this meta-analysis, combined with future research, should prompt the consideration of including exercise in alcohol treatment in inpatient, outpatient, and community settings.

## Data Availability Statement

The original contributions presented in the study are included in the article/supplementary material, further inquiries can be directed to the corresponding author/s.

## Author Contributions

DL, KC, and MZ contributed to conception and design of the study. DL, KC, KH, FA, and MZ engaged in review of manuscripts for meta-analysis and wrote sections of manuscript. DL and MZ conducted meta-analyses. All authors contributed to manuscript revision, read, and approved the submitted version.

## Conflict of Interest

The authors declare that the research was conducted in the absence of any commercial or financial relationships that could be construed as a potential conflict of interest.

## References

[B1] Åhlin J. Hallgren M Öjehagen, A. Källmén H. Forsell Y. (2015). Adults with mild to moderate depression exhibit more alcohol related problems compared to the general adult population: a cross sectional study. BMC Public Health 15, 1–7. 10.1186/s12889-015-1837-8 26051511 PMC4459061

[B2] Ahlskog J. E. Geda Y. E. Graff-Radford N. R. Petersen R. C. (2011). Physical exercise as a preventive or disease-modifying treatment of dementia and brain aging. Mayo Clin. Proc. 86, 876–884. 10.4065/mcp.2011.0252 21878600 PMC3258000

[B3] American Psychiatric Association (2013). Diagnostic and Statistical Manual of Mental Disorders (DSM-5®). Washington, DC: American Psychiatric Publication. 10.1176/appi.books.9780890425596

[B4] Berglund M. Thelander S. Salaspuro M. Franck J. Andréasson S. Öjehagen A. (2003). Treatment of alcohol abuse: an evidence-based review. Alcohol. Clin. Exp. Res. 27, 1645–1656. 10.1002/3527601465 14574236

[B5] Brown R. A. Abrantes A. M. Minami H. Read J. P. Marcus B. H. Jakicic J. M.. (2014). A preliminary, randomized trial of aerobic exercise for alcohol dependence. J. Sub. Abuse Treat. 47, 1–9. 10.1016/j.jsat.2014.02.004 24666811 PMC4648239

[B6] Brown R. A. Prince M. A. Minami H. Abrantes A. M. (2016). An exploratory analysis of changes in mood, anxiety and craving from pre-to post-single sessions of exercise, over 12 weeks, among patients with alcohol dependence. Mental Health Phys. Activity 11, 1–6. 10.1016/j.mhpa.2016.04.002 29606975 PMC5878084

[B7] Cabe N. Laniepce A. Pitel A. L. (2020). Physical activity and promising adjunctive treatment for severe alcohol use disorder. Add. Behav. 2020:106667. 10.1016/j.addbeh.2020.106667 33074123

[B8] Carroll K. M. Onken L. S. (2005). Behavioral therapies for drug abuse. Am. J. Psychiatry 162, 1452–1460. 10.1176/appi.ajp.162.8.1452 16055766 PMC3633201

[B9] Carvalho A. F. Heilig M. Perez A. Probst C. Rehm J. (2019). Alcohol use disorders. Lancet 394, 781–792. 10.1016/S0140-6736(19)31775-1 31478502

[B10] Castillo-Carniglia A. Keyes K. M. Hasin D. S. Cerdá M. (2019). Psychiatric comorbidities in alcohol use disorder. Lancet Psychiatry 6, 1068–1080. 10.1016/S2215-0366(19)30222-6 31630984 PMC7006178

[B11] Centers of Disease Control and Prevention. (2020). “Binge drinking,” in Centers for Disease Control and Prevention: Centers for Disease Control and Prevention (Washington, DC).

[B12] Davis-Martin R. E. Alessi S. M. Boudreaux E. D. (2021). Alcohol use disorder in the age of technology: a review of wearable biosensors in alcohol use disorder treatment. Front. Psychiatry 12:246. 10.3389/fpsyt.2021.642813 33828497 PMC8019775

[B13] Di Nicola M. Tedeschi D. De Risio L. Pettorruso M. Martinotti G. Ruggeri F.. (2015). Co-occurrence of alcohol use disorder and behavioral addictions: relevance of impulsivity and craving. Drug Alcohol Depend. 148, 118–125. 10.1016/j.drugalcdep.2014.12.028 25630963

[B14] DiClemente C. C. Carbonari J. P. Montgomery R. Hughes S. O. (1994). The alcohol abstinence self-efficacy scale. J. Stud. Alcohol 55, 141–148. 10.15288/jsa.1994.55.141 8189734

[B15] Flannery B. Volpicelli J. Pettinati H. (1999). Psychometric properties of the Penn alcohol craving scale. Alcoholism 23, 1289–1295. 10.1111/j.1530-0277.1999.tb04349.x 10470970

[B16] Fleury M.-J. Djouini A. Huỳnh C. Tremblay J. Ferland F. Ménard J.-M.. (2016). Remission from substance use disorders: a systematic review and meta-analysis. Drug Alcohol Depend. 168, 293–306. 10.1016/j.drugalcdep.2016.08.625 27614380

[B17] Frascella J. Potenza M. N. Brown L. L. Childress A. R. (2010). Shared brain vulnerabilities open the way for nonsubstance addictions: carving addiction at a new joint? Ann. N. Y. Acad. Sci. 1187, 294–315. 10.1111/j.1749-6632.2009.05420.x 20201859 PMC3671907

[B18] Furzer B. Rebar A. Dimmock J. A. More A. Thornton A. L. Wright D.. (2021). Exercise is medicine… when you enjoy it: exercise enjoyment, relapse prevention efficacy, and health outcomes for youth within a drug and alcohol treatment service. Psychol. Sport Exerc. 52:101800. 10.1016/j.psychsport.2020.101800

[B19] Gates P. Albertella L. (2016). The effectiveness of telephone counselling in the treatment of illicit drug and alcohol use concerns. J. Telemed. Telecare 22, 67–85. 10.1177/1357633X15587406 26026185

[B20] Glozah F. N. Adu N. A. T. Komesuor J. (2015). Assessing alcohol abstinence self-efficacy in undergraduate students: psychometric evaluation of the alcohol abstinence self-efficacy scale. Health Q. Life Outcomes 13:189. 10.1186/s12955-015-0387-1 26607755 PMC4659213

[B21] Greenwood B. N. Loughridge A. B. Sadaoui N. Christianson J. P. Fleshner M. (2012). The protective effects of voluntary exercise against the behavioral consequences of uncontrollable stress persist despite an increase in anxiety following forced cessation of exercise. Behav. Brain Res. 233, 314–321. 10.1016/j.bbr.2012.05.017 22610051 PMC3402647

[B22] Gür F. Gür G. C. (2020). Is Exercise a Useful Intervention in the Treatment of Alcohol Use Disorder? Systematic Review and Meta-Analysis. AJHP 34, 520–537. 10.1177/0890117120913169 32212949

[B23] Hallgren M. Ahlin J. Forsell Y. Öjehagen A. (2014a). Increased screening of alcohol habits among patients with depression is needed. Scand. J. Public Health 42, 658–659. 10.1177/1403494814551860 25269790

[B24] Hallgren M. Romberg K. Bakshi A.-S. Andréasson S. (2014b). Yoga as an adjunct treatment for alcohol dependence: a pilot study. Compl. Therap. Med. 22, 441–445. 10.1016/j.ctim.2014.03.003 24906582

[B25] Hallgren M. Vancampfort D. Giesen E. S. Lundin A. Stubbs B. (2017a). Exercise as treatment for alcohol use disorders: systematic review and meta-analysis. Br. J. Sports Med. 51, 1058–1064. 10.1136/bjsports-2016-096814 28087569

[B26] Hallgren M. Vancampfort D. Schuch F. Lundin A. Stubbs B. (2017b). More reasons to move: exercise in the treatment of alcohol use disorders. Front. Psychiatry 8:160. 10.3389/fpsyt.2017.00160 28894426 PMC5581356

[B27] Hedges L. V. Olkin I. (2014). Statistical Methods for Meta-Analysis. Academic press (2014).

[B28] Heggelund J. Vancampfort D. Tacchi M. J. Morken G. Scott J. (2020). Is there an association between cardiorespiratory fitness and stage of illness in psychotic disorders? A systematic review and meta-analysis. Acta psych. Scand. 141, 190–205. 10.1111/acps.13119 31646608

[B29] Higgins J. P. Thomas J. Chandler J. Cumpston M. Li T. Page M. J.. (2019). Cochrane Handbook for Systematic Reviews of Interventions. Hoboken, NJ: John Wiley & Sons. 10.1002/9781119536604 PMC10284251 31643080

[B30] Jennison K. M. (2004). The short-term effects and unintended long-term consequences of binge drinking in college: a 10-year follow-up study. Am. J. Drug Alcohol Abuse 30, 659–684. 10.1081/ADA-200032331 15540499

[B31] Jensen C. D. Cushing C. C. Aylward B. S. Craig J. T. Sorell D. M. Steele R. G. (2011). Effectiveness of motivational interviewing interventions for adolescent substance use behavior change: a meta-analytic review. J. Consult. Clin. Psychol. 79:433. 10.1037/a0023992 21728400

[B32] Jensen K. Nielsen C. Ekstrøm C. T. Roessler K. K. (2019). Physical exercise in the treatment of alcohol use disorder (AUD) patients affects their drinking habits: a randomized controlled trial. Scand. J. Public Health 47, 462–468. 10.1177/1403494818759842 29480087

[B33] Jhanjee S. (2014). Evidence based psychosocial interventions in substance use. Indian J. Psychol. Med. 36, 112–118. 10.4103/0253-7176.130960 24860208 PMC4031575

[B34] Jin H. Rourke S. B. Patterson T. L. Taylor M. J. Grant I. (1998). Predictors of relapse in long-term abstinent alcoholics. J. Studies Alcohol 59, 640–646. 10.15288/jsa.1998.59.640 9811085

[B35] Kendler K. S. Sundquist K. Ohlsson H. Palmér K. Maes H. Winkleby M. A. Sundquist J. (2012). Genetic and familial environmental influences on the risk for drug abuse: a national Swedish adoption study. Arch. Gen. Psychiatry. 69, 690–697. 10.1001/archgenpsychiatry.2011.2112 22393206 PMC3556483

[B36] Knapp W. P. Soares B. Farrell M. Silva de Lima M. (2016). Psychosocial interventions for cocaine and psychostimulant amphetamines related disorders. Cochrane. Database. Syst. Rev. 3:CD003023. 10.1002/14651858.CD003023.pub2 25835305 PMC10687506

[B37] Lai H. M. X. Cleary M. Sitharthan T. Hunt G. E. (2015). Prevalence of comorbid substance use, anxiety and mood disorders in epidemiological surveys, 1990–2014: a systematic review and meta-analysis. Drug Alcohol Depend. 154, 1–13. 10.1016/j.drugalcdep.2015.05.031 26072219

[B38] Lipari R. N. Park-Lee E. Van Horn S. (2016). “America's need for and receipt of substance use treatment in 2015,” in The CBHSQ Report. Substance Abuse and Mental Health Services Administration (US). 28080008

[B39] Lynch W. J. Peterson A. B. Sanchez V. Abel J. Smith M. A. (2013). Exercise as a novel treatment for drug addiction: a neurobiological and stage-dependent hypothesis. Neurosci. Biobehav. Rev. 37, 1622–1644. 10.1016/j.neubiorev.2013.06.011 23806439 PMC3788047

[B40] Maisto S. A. Connors G. J. Zywiak W. H. (2000). Alcohol treatment changes in coping skills, self-efficacy, and levels of alcohol use and related problems 1 year following treatment initiation. Psychol. Addict. Behav. 14:257. 10.1037/0893-164X.14.3.257 10998951

[B41] Marazziti D. Baroni S. (2014). EPA-1745–Plasma clomipramine levels in adult obsessive-compulsive disorder patients. Europ. Psychiatry 29, 1–1. 10.1016/S0924-9338(14)78876-5 21979789

[B42] Mattson M. Allen J. Miller W. Hester R. Connors G. Rychtarik R.. (1993). Project MATCH: Rationale and methods for a multisite clinical trial matching patients to alcoholism treatment. Alcoholism. Clin. Exp. Res. 17, 1130–1145. 10.1111/j.1530-0277.1993.tb05219.x 8116822

[B43] McKercher C. Sanderson K. Schmidt M. D. Otahal P. Patton G. C. Dwyer T.. (2014). Physical activity patterns and risk of depression in young adulthood: a 20-year cohort study since childhood. Soc. Psychiatry Psychiatr. Epidem. 49, 1823–1834. 10.1007/s00127-014-0863-7 24626994

[B44] Miller K. J. Areerob P. Hennessy D. Gonçalves-Bradley D. C. Mesagno C. Grace F. (2020). Aerobic, resistance, and mind-body exercise are equivalent to mitigate symptoms of depression in older adults: a systematic review and network meta-analysis of randomised controlled trials. F1000Research 9:1325. 10.12688/f1000research.27123.1 34158928 PMC8191520

[B45] Miller W. R. Walters S. T. Bennett M. E. (2001). How effective is alcoholism treatment in the United States? J. Studies Alcohol 62, 211–220. 10.15288/jsa.2001.62.211 11327187

[B46] Mitchell C. McLanahan S. Brooks-Gunn J. Garfinkel I. Hobcraft J. Notterman D. (2013). Genetic differential sensitivity to social environments: Implications for researcher. Am. J. Public Health 103, S102–S110. 10.2105/AJPH.2013.301382 23927507 PMC4012542

[B47] Moher D. Liberati A. Tetzlaff J. Altman D. G. (2009). Preferred reporting items for systematic reviews and meta-analyses: the PRISMA statement. PLoS Med. 6:e1000097. 10.1371/journal.pmed1000097 19621072 PMC2707599

[B48] Mojtabai R. Chen L.-Y. Kaufmann C. N. Crum R. M. (2014). Comparing barriers to mental health treatment and substance use disorder treatment among individuals with comorbid major depression and substance use disorders. J. Subst. Abuse Treatment 46, 268–273. 10.1016/j.jsat.2013.07.012 23992953 PMC3840086

[B49] Moos R. H. Moos B. S. (2006). Participation in treatment and alcoholics anonymous: a 16-year follow-up of initially untreated individuals. J. Clin. Psych. 62, 735–750. 10.1002/jclp.20259 16538654 PMC2220012

[B50] Moos R. H. Moos B. S. Finney J. W. (2001). Predictors of deterioration among patients with substance-use disorders. J. Clin. Psych. 57, 1403–1419. 10.1002/jclp.1105 11745584

[B51] Moos R. H. Nichol A. C. Moos B. S. (2002). Risk factors for symptom exacerbation among treated patients with substance use disorders. Addiction 97, 75–85. 10.1046/j.1360-0443.2002.00063.x 11895273

[B52] Murphy T. J. Pagano R. R. Marlatt G. A. (1986). Lifestyle modification with heavy alcohol drinkers: effects of aerobic exercise and meditation. Add. Behav. 11, 175–186. 10.1016/0306-4603(86)90043-2 3526824

[B53] Musselman J. R. Rutledge P. C. (2010). The incongruous alcohol-activity association: physical activity and alcohol consumption in college students. Psychol. Sport Exerci. 11, 609–618. 10.1016/j.psychsport.2010.07.005

[B54] National Institute on Alcohol Abuse Alcoholism (2017). “Alcohol facts and statistics,” in NIAAA. Washington, DC: National Institutes of Health, US Department of Health and Human.

[B55] Nelson T. F. Wechsler H. (2001). Alcohol and college athletes. Med Sci Sports Exerc. 33, 43–47. 10.1097/00005768-200101000-00008 11194110

[B56] Powell K. G. Treitler P. Peterson N. A. Borys S. Hallcom D. (2019). Promoting opioid overdose prevention and recovery: an exploratory study of an innovative intervention model to address opioid abuse. Int. J. Drug Policy 64, 21–29. 10.1016/j.drugpo.2018.12.004 30551002

[B57] Roessler K. K. Bilberg R. Søgaard Nielsen A. Jensen K. Ekstrøm C. T. Sari S. (2017). Exercise as adjunctive treatment for alcohol use disorder: a randomized controlled trial. PLoS ONE 12:e0186076. 10.1371/journal.pone.0186076 29049336 PMC5648142

[B58] Ruegsegger G. N. Booth F. W. (2018). Health benefits of exercise. Cold Spring Harbor Persp. Med. 8:a029694. 10.1101/cshperspect.a029694 PMC6027933 28507196

[B59] Seneviratne C. Johnson B. A. (2015). Advances in medications and tailoring treatment for alcohol use disorder. Alcohol Res. Curr. Rev. 37:15. 26259086 10.35946/arcr.v37.1.02 PMC4476601

[B60] Shaw G. Waller S. Latham C. Dunn G. Thomson A. (1997). Alcoholism: A long-term follow-up study of participants in an alcohol treatment programme. Alcohol Alcohol. 32, 527–535. 10.1093/oxfordjournals.alcalc.a008288 9269861

[B61] Simpson D. D. Sells S. B. (1982). Effectiveness of treatment for drug abuse: an overview of the DARP research program. Adv. Alcohol Subst. Abuse 2, 7–29. 10.1300/J251v02n01_02

[B62] Sinyor D. Brown T. Rostant L. Seraganian P. (1982). The role of a physical fitness program in the treatment of alcoholism. J. Stud. Alcohol 43, 380–386. 10.15288/jsa.1982.43.380 7121004

[B63] Taylor A. H. Oh H. Cullen S. (2013). Acute effect of exercise on alcohol urges and attentional bias towards alcohol related images in high alcohol consumers. Mental Health Physical Activity 6, 220–226. 10.1016/j.mhpa.2013.09.004

[B64] Thompson S. G. Higgins J. P. (2002). How should meta-regression analyses be undertaken and interpreted? Statist. Med. 21, 1559–1573. 10.1002/sim.1187 12111920

[B65] Thompson T. Oram C. Correll C. U. Tsermentseli S. Stubbs B. (2017). Analgesic effects of alcohol: a systematic review and meta-analysis of controlled experimental studies in healthy participants. J. Pain 18, 499–510. 10.1016/j.jpain.2016.11.009 27919773

[B66] van Rhee H. Suurmond R. Hak T. (2015). User Manual for Meta-Essentials: Workbooks for Meta-Analysis. Available online at: SSRN 3241355. 10.2139/ssrn.3241355

[B67] Vancampfort D. Hallgren M. Mugisha J. Hert M. D. Probst M. Monsieur D.. (2016). The prevalence of metabolic syndrome in alcohol use disorders: a systematic review and meta-analysis. Alcohol Alcoh. 51, 515–521. 10.1093/alcalc/agw040 27337988

[B68] Verheul R. van den Brink W. Geerlings P. (1999). A three-pathway psychobiological model of craving for alcohol. Alcohol Alcoh. 34, 197–222. 10.1093/alcalc/34.2.197 10344781

[B69] Vickers K. S. Patten C. A. Bronars C. Lane K. Stevens S. R. Croghan l. T.. (2004). Binge drinking in female college students: the association of physical activity, weight concern, and depressive symptoms. J. Am. College Health 53, 133–140. 10.3200/JACH.53.3.133-140 15571116

[B70] Weinstock J. Capizzi J. Weber S. M. Pescatello L. S. Petry N. M. (2014). Exercise as an intervention for sedentary hazardous drinking college students: a pilot study. Mental Health Phys. Activity 7, 55–62. 10.1016/j.mhpa.2014.02.002 24949085 PMC4058428

[B71] Weinstock J. Petry N. M. Pescatello L. S. Henderson C. E. Nelson C. R. (2020). Randomized clinical trial of exercise for nontreatment seeking adults with alcohol use disorder. Psychol. Addict. Behav. 34:65. 31424244 10.1037/adb0000506 PMC7007324

[B72] Wendt D. C. Gone J. P. (2017). Group therapy for substance use disorders: a survey of clinician practices. J. Groups Addict. Recov. 12, 243–259. 10.1080/1556035X.2017.1348280 30546274 PMC6289265

[B73] Witkiewitz K. Litten R. Leggio L. (2019). Advances in the science and treatment of alcohol use disorder. Sci. Adv. 5:eaax4043. 10.1126/sciadv.aax4043 31579824 PMC6760932

[B74] World Health Organization. (2010). Global Strategy to Reduce the Harmful Use of Alcohol. Geneva: World Health Organization. 10.2471/BLT.19.241737 PMC7047030 32132758

